# Mutation of *Serine protease 1* Induces Male Sterility in *Bombyx mori*

**DOI:** 10.3389/fphys.2022.828859

**Published:** 2022-02-10

**Authors:** Xia Xu, Yaohui Wang, Jine Chen, Xin Du, Lusong Yao, Jun Xu, Yong Zhang, Yongping Huang, Yongqiang Wang

**Affiliations:** ^1^Institute of Sericulture and Tea, Zhejiang Academy of Agricultural Sciences, Hangzhou, China; ^2^Key Laboratory of Insect Developmental and Evolutionary Biology, CAS Center for Excellence in Molecular Plant Sciences, Shanghai Institute of Plant Physiology and Ecology, Chinese Academy of Sciences, Shanghai, China

**Keywords:** *BmSer1*, DNA molecule mutation, male sterility, *Bombyx mori*, CRISPR/Cas9

## Abstract

Serine proteases are important in reproduction, embryonic development, cell differentiation, apoptosis, and immunity. The genes encoding some serine proteases are essential for male fertility in both humans and rodents and are functionally conserved among metazoan. For example, the *Serine protease 1* (*Ser1*) gene determines male reproductive success in the model lepidopteran insect *Bombyx mori*. In this study, we explored the function of *BmSer1* through transgenic CRISPR/Cas9 technology-mediated mutations in silkworm. We found that the mutation of *BmSer1* gene resulted in male sterility but had no effect on female fertility. Male mutants produce normal eupyrene sperm bundles, but the sperm bundles do not dissociate into single sperm. Male sterility caused by the *BmSer1* gene mutation was inherited stably through female individuals. Therefore, the serine protease encoded by *BmSer1* is essential for male reproductive success in lepidopterans and is a potential target gene for biological reproductive regulation.

## Introduction

Serine proteases are important proteolytic enzymes that have serine as the active center. More than one-third of known proteolytic enzymes are serine proteases (Page and Di Cera, 2008). Serine proteases are divided into three categories based on substrate specificity: chymotrypsin, trypsin, and elastase (Kasafirek et al., 1976). These proteases are mainly β-proteins in structure, and their sequences have diverged greatly during evolution, but the active sites all contain three amino acid residues of Ser, His, and Asp (Wallace et al., 1996; Betzel et al., 2001). His and Asp are located in the N-terminal domain and ensure structural stability and functional activity, whereas Ser is located in the C-terminal domain and has catalytic function (Kraut, 1977; Zhou et al., 1994). Serine proteases are important for reproduction, embryonic development, cell differentiation, apoptosis, and immunity in animals (Liu et al., 2004; Bhuiyan and Fukunaga, 2008; Wang et al., 2008; Lin et al., 2015; Lee et al., 2018; Barzkar et al., 2021). Loss or deficiency of serine proteases can lead to severe development defects and sterility (Liu et al., 2013; Shang et al., 2018; Holcomb et al., 2020; Kobayashi et al., 2020). In recent years, a considerable number of studies have showed serine proteases are specifically expressed in reproductive tissues in *Bombyx mori* (Cesari et al., 2010). For example, we have successfully characterized and functionally ovarian serine protein or egg specific protein, as primary proteins conferring the oogenesis and fertility in *B. mori* (Xu et al., 2020b,^2021^).

The complex physiological process of spermatogenesis, which involves mitosis, meiosis, and morphological changes, is also regulated by serine proteases that are essential for reproductive success (Le Magueresse-Battistoni, 2007; LaFlamme and Wolfner, 2013; Salicioni et al., 2020). The trypsin-like serine protease 37 (PRSS37) is highly and exclusively expressed in the testis of adult mouse, especially in elongating spermatids during spermatogenesis. Loss of PRSS37 expression cause defective sperm migration from the uterus into oviduct, resulting in male infertility in humans and mouses (Liu et al., 2016; Xiong et al., 2021). Deletion of the gene encoding the serine protease PCSK4 in mouse leads to accelerated capacitation of sperm, impaired binding of sperm to zona pellucida, impaired fertilization, and ultimately infertility (Gyamera-Acheampong and Mbikay, 2009; Tardif et al., 2012). The testis-specific serine kinase family (TSSK) has six members, which are all expressed post-meiotically during spermiogenesis. The members of the TSSK family have high homologies in their kinase domains, and their defects lead to sterility without exhibiting somatic abnormalities (Nayyab et al., 2021). In *Drosophila melanogaster*, the serine protease Seminase acts as seminal fluid protein component and initiates protease cascade signaling pathway through hydrolysis, thus participating in early post-copulation reproductive regulation. RNA interference (RNAi) technology was used to down-regulate the expression of the *Seminase* gene, resulting in reduced oviposition and other sex peptide storage defects (LaFlamme et al., 2012). In the silkworm *B. mori* and *Plutella xylostella*, the serine protease 2 is a component of seminal fluid, and reductions in its expression lead to male sterility (Xu et al., 2020c).

In addition to the serine protease 2, there are many other serine proteases in the seminal fluid (Le Magueresse-Battistoni, 2007; LaFlamme and Wolfner, 2013; Salicioni et al., 2020). Confusingly, why these serine proteases are necessary and what are the functions of the different proteases? On the other hand, the function of serine protease in reproduction has been studied, but there are few reports in the model lepidopteran insect *B. mori*. Lepidoptera, the second largest insect order containing more than 70% of the existing agroforestry insect pests (Roscoe et al., 2016). In order to explore the function of other serine proteases in silkworm for more potential sterile gene targets, we here investigated the function of *serine protease 1* (*Ser1*) (NM_001160202.1) in *B. mori* using transgenic CRISPR/Cas9 technology. We found that the loss of function of *BmSer1* resulted in male sterility without reducing female fertility and without affecting growth and development. In bursa copulatrix of females mated with male mutants, due to the eupyrene sperm bundles failed to dissociate into single sperm, resulting in subsequent fertilization failures. Importantly, the male sterility phenotypes were inherited stably to offspring of female mutants that carried the *BmSer1* male sterility gene mutations. The competitiveness of mutants was the same as that of the wild-type insects, and the *BmSer1* is moderately conserved in evolution. Taken together, our data indicate that the *Ser1* gene has potential as a genetic-based inheritable sterile insect technology (SIT) for pest control.

## Materials and Methods

### Insect Strains and Rearing

Nistari, a multivoltine and non-diapausing silkworm strain, was used for all experiments. Larvae were reared on fresh mulberry leaves at 25°C under standard conditions (Tan et al., 2005).

### Protein Structure Analysis

Protein structure was modeled using the online software SWISS-MODEL^1^ (Bienert et al., 2017). Visual Molecular Dynamics software was used for visual analysis (Giorgino, 2019).

### Phylogenetic Analysis

Evolutionary history was inferred using the neighbor-joining method (Saitou and Nei, 1987). The evolutionary distances were computed using the Poisson correction method and are in the units of the number of amino acid substitutions per site. The analysis involved amino acid sequences of the Ser1 homologs from *B. mori*, *Ostrinia furnacalis*, *Papilio xuthus*, *Spodoptera litura*, *Helicoverpa armigera*, *Operophtera brumata*, *Heliconius melpomene*, *Eueides isabella*, *Bicyclus anynana*, and *Laparus doris*. All ambiguous positions were removed for each sequence pair (pairwise deletion option). Evolutionary analyses were conducted in MEGA X (Kumar et al., 2018).

### RNA Isolation, Complementary DNA Synthesis, and qPCR Analysis

Total RNA was isolated from several silkworm tissues using TRIzol^®^ reagent (Invitrogen, United States). For complementary DNA (cDNA) synthesis, 1 μg of total RNA was used with the RevertAid™ First Strand cDNA Synthesis Kit (Thermo Fisher Scientific, United States). Quantitative real-time PCR (qRT-PCR) analyses were performed using a SYBR Green Realtime PCR Master Mix (Thermo Fisher Scientific, United States). The PCR conditions were as follows: initial incubation at 95°C for 5 min, 35 cycles at 95°C for 15 s, and 60°C for 1 min. The *B. mori* gene encoding ribosomal protein 49 (*Bmrp49*) was used as an internal control. A relative quantitative method (^△△^Ct) was used to evaluate quantitative variation. The gene-specific primers used for qRT-PCR are listed in Table 1.

**TABLE 1:** Primers used in PCR amplification and plasmid construction.

Primer name	Primer sequence (5′–3′)
**qRT-PCR analysis**	
BmSer1-F	ATTCTGGTGCGATGAAAGGC
BmSer1-R	TCACCCTGGCACGTATCTTT
Bmrp49-F	TCAATCGGATCGCTATGACA
Bmrp49-R	ATGACGGGTCTTCTTGTTGG

**Plasmid construction**	
BmSer1-U6-F	CTCACTATAGGGCGAATTGGAGGTTATGTAGTACACATT GTTGTA
BmSer1-U6-R	TTTTCTTGTTATAGATATCAAAAAAAGCACCGACTCGGTG
BmSer1-Overlap-F	GCTAGCCATTGACTCCGCGGAGGTTATGTAGTACACATT GTTGTA
BmSer1-Overlap-R	CCGCGGAGTCAATGGCTAGCAAAAAAGCACCGACTCG GTG
BmSer1-sg1-F	GGTCCTCATTGGGTTTAGAAGTTTTAGAGCTAGAAATAG CAAGTT
BmSer1-sg1-R	TTCTAAACCCAATGAGGACCACTTGTAGAGCACGATATT TTGTAT
BmSer1-sg2-F	GGCGGTAAAGATACGTGCCAGTTTTAGAGCTAGAAATAG CAAGTT
BmSer1-sg2-R	TGGCACGTATCTTTACCGCCACTTGTAGAGCACGATATT TTGTAT

**Identification of mutations**	
BmSer1-KO-F	GTGGGAGTACTATTACGGGAAG
BmSer1-KO-R	CTATCGATAAACGTAGCGGCGT

### Plasmid Construction

The sgRNA sequences that matched the 5′-GG-N18-NGG-3′ rule were identified and potential off-target binding to the relevant silkworm genomic sequence was analyzed using CRISPRdirect^2^ (Naito et al., 2015). Two 23-base-pair (bp) sgRNAs that target sites in the exon of *Ser1* were designed. The activator was the plasmid *pBac[IE1-EGFPNos-Cas9]* (*Nos-Cas9*), which results in Cas9 expression, driven by the *nanosP* (*Nos*) promoter located in the posterior region of embryos near the gonad site (Xu et al., 2019); and expression of the enhanced green fluorescent protein (EGFP) marker under control of the *IE1* promoter as reported in our previous study (Nakao et al., 2008; Xu et al., 2019, 2020a). The effector plasmid was *pBac[IE1-DsRed2-U6-sgRNA]* (*U6-sgRNA*), which results in expression of the sgRNA under control of the silkworm *U6* promoter and the DsRed fluorescence marker under control of the *IE1* promoter. The primers used for plasmid construction are listed in Table 1.

### Silkworm Germline Transformation and Mutagenesis Analysis

Silkworm germline transformation was performed by microinjection of a mixed solution of *U6-sgRNA* and *piggyBac* helper vector into pre-blastoderm Nistari embryos. Embryos were incubated in a humidified chamber at 25°C until hatching. Larvae were reared to moths and sib-mated or backcrossed with wild-type (WT) moths. G1 progeny were scored for the presence of the marker gene during the embryonic stage under a fluorescence microscope (Nikon AZ100, Japan). Four germlines were produced by the hybridization of the *Nos-Cas9* line with the *U6-sgRNA* line were the F1 progeny, including the mutant line with double-fluorescences (DsRed and EGFP), *Nos-Cas9* line with green fluorescence (EGFP), the *U6-sgRNA* line with red fluorescence (DsRed), and a non-mutant line without fluorescence. Free hybridization of four lines also produced only these four types of individuals. The inheritance of double-fluorescences was confirmed in each subsequent generation. Individuals with double-fluorescences (DsRed and GFP) were Δ*BmSer1* somatic mutants and were used in subsequent experiments.

Genomic DNA of Δ*BmSer1* individuals was extracted to identify deleted regions. First instar larvae were incubated in DNA extraction buffer (1:1:2:2.5 ratio of 10% SDS to 5 M NaCl to 100 mM EDTA to 500 mM Tris-HCl, pH 8) with proteinase K, then purified with a standard phenol:chloroform extraction and isopropanol precipitation, followed by RNaseA treatment. The genomic PCR conditions were as follows: 94°C for 2 min, 35 cycles of 94°C for 15 s, 55°C for 30 s, and 72°C for 1 min, followed by a final extension period at 72°C for 10 min. The PCR products were sub-cloned into pJET1.2 vectors (Thermo Fisher Scientific, United States), and vectors were sequenced. The primers used for sequencing are listed in Table 1.

### Sperm Morphology Observation

Bursa copulatrixes were dissected from mated control and Δ*BmSer1* individuals. The tissues were prefixed with 4% paraformaldehyde fixative (Beyotime, CHN), placed on a glass slide, and photographed under a microscope (Olympus BX51, Japan).

### Mutant Competitiveness and Germline Transmission Assay

A competitiveness assay was performed in a plastic container (30 × 18 × 4.5 cm^3^). To evaluate mutant females, newly emerged WT and Δ*BmSer1* female moths were placed on either side of the container leaving a distance of 10 cm to the center, and one newly emerged WT male moth was placed in the center of the container. To evaluate mutant males, newly emerged WT and Δ*BmSer1* male moths were placed on either side of the container, and a newly emerged WT female moth was placed in the center of the container. Male moths that mated with a female were considered responsive, and the number was recorded. The response index was calculated as a percentage of the number of responsive moths compared with the total number of test moths.

### Statistical Analysis

Three independent replicates were used for each treatment. Means were determined, and error bars are ± SEM. A two-tailed Student’s *t*-test was used to analyze differences between WT and mutant individuals.

## Results

### Protein Structure and Phylogenetic Identification of Ser1

**FIGURE 1:**
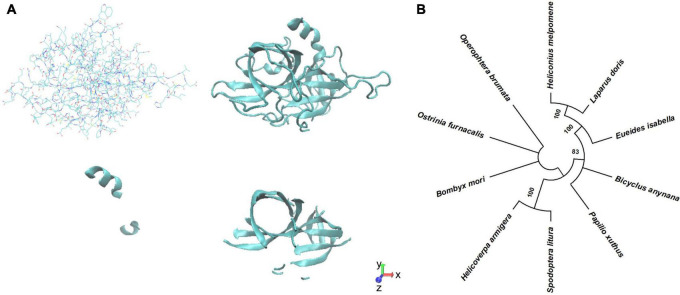
Predicted structure Phylogenetic analysis of BmSer1 protein. **(A)** Predicted structure in licorice representation and ribbon diagram. **(B)** Phylogenetic tree. Protein sequences from *B. mori* (NP_001153674.1), *O. furnacalis* (XP_028171578.1), *P. xuthus* (XP_013174764.1), *S. litura* (XP_022835461.1), *H. armigera* (XP_021184761.1), *O. brumata* (KOB66629.1), *H. melpomene* (ADJ58567.1), *E. isabella* (AEU11620.1), *B. anynana* (XP_023944151.1), *and L. doris* (AEU11625.1). The tree is drawn to scale, with branch lengths in the same units as those of the evolutionary distances used to infer the phylogenetic tree.

We modeled the structure of BmSer1 protein using SWISS-MODEL, and visualized the model by Visual Molecular Dynamics software. The protein is predicted to fold into 2 α-helices and 14 β-sheets (Figure 1A). Homologous sequences of the Ser1 protein were selected from 10 different lepidoptera species to explore evolutionary conservation. These species were *B. mori*, *Ostrinia furnacalis*, *Papilio xuthus*, *Spodoptera litura*, *Helicoverpa armigera*, *Operophtera brumata*, *Heliconius melpomene*, *Eueides isabella*, *Bicyclus anynana*, and *Laparus doris*. The Ser1 protein sequence is moderately conserved (Figure 1B). Most serine proteases are highly specific for certain substrates, and therefore it is likely that the serine proteases involved in reproduction are species specific. We speculate that serine protease specificity may be a factor in reproductive isolation between different organisms. The specificity of serine proteases has not yet been elucidated.

### Spatiotemporal Expression Pattern of *BmSer1*

**FIGURE 2:**
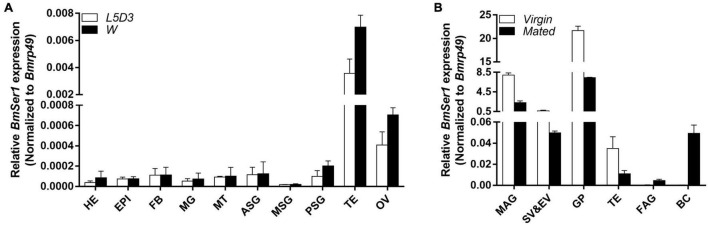
Spatial and temporal expression of *BmSer1* in WT individuals. **(A)** *BmSer1* expression in ten tissues at L5D3 and W stages. **(B)** *BmSer1* expression in six tissues at virgin and mated adult stages. Abbreviations: epidermis (EPI), fat body (FB), midgut (MG), Malpighian tubules (MT), anterior silk gland (ASG), middle silk gland (MSG), posterior silk gland (PSG), testis (TE), ovary (OV), male accessory gland (MAG), seminal vesicle and ejaculatory vesicle (SV&EV), glandula prostatica (GP), female accessory gland (FAG), and bursa copulatrix (BC). mRNA expression was normalized to *Bmrp49*. The data shown are means ± S.E.M.

We investigated the transcriptional profile of *BmSer1* from two developmental stages: day 3 of the fifth instar larvae (L5D3) and the wandering stage (W). For each developmental stage, we collected 10 different tissues for qRT-PCR analysis: head, epidermis, fat body, midgut, Malpighian tubules, anterior silk gland, middle silk gland, posterior silk gland, testis, and ovary. The results showed that *BmSer1* was more highly expressed in the testis than other tissues in both L5D3 and W stages (Figure 2A). Since *BmSer1* is highly expressed in the larval testis, we subsequently examined its expression in the adult gonads. We quantified the *BmSer1* mRNA expression in six major reproductive tissues of virgin and mated adults, including male accessory gland, seminal vesicle and ejaculatory vesicle, glandula prostatica, testis, female accessory gland, and bursa copulatrix. *BmSer1* was more highly expressed in the glandula prostatica than other tissues in both virgin and mated adult stages (Figure 2B). These results suggested that the *BmSer1* might be important in male fertility.

### CRISPR/Cas9-Mediated Mutagenesis

**FIGURE 3:**
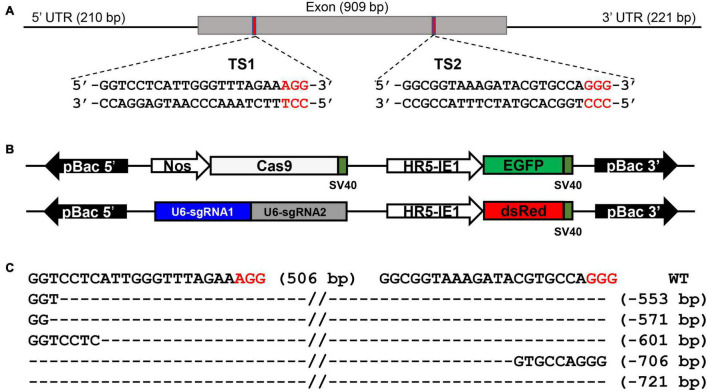
*BmSer1* knockout mediated by transgenic CRISPR/Cas9 system. **(A)** Genomic structure of *BmSer1*. The locations and sequences of the binding sites for sgRNAs (TS1 and TS2) in the exon of *BmSer1* are indicated. **(B)** Schematic representations the activator line vector *Nos-Cas9* and effector line vector *U6-sgRNA*. **(C)** Mutations observed in representative heterozygous offspring. The target sequence is in black, and the PAM sequences are in red. The deletion size is indicated to the right of the sequence.

We generated *BmSer1* loss-of-function silkworms using the *CRISPR/Cas9*. The *BmSer1* gene consists of only one exon, and we selected two regions to target with sgRNAs that fit the consensus GGN_19_GG (Figure 3A). The *BmSer1* mutants were obtained by crossing the strain *Nos-Cas9*, which encodes Cas9 (with EGFP as a selection marker), with the U6-sgRNA strain that encodes the *BmSer1*-targeted sgRNAs (with DsRed as a selection marker) (Figure 3B). The silkworm mutants were obtained from the progenies carrying both EGFP and DsRed fluorescent markers. The mutation events were confirmed by genomic PCR. All the mutations of *BmSer1* in the transgenic line were somatic mutations, so the mutation types were varied (Figure 3C).

### *BmSer1* Mutation Induce Male Sterility

**FIGURE 4:**
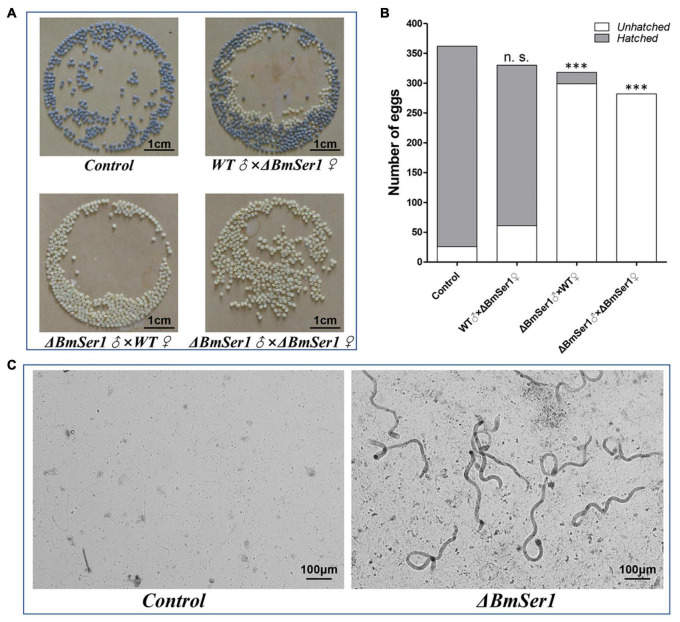
*BmSer1* mutations result in male sterility. **(A)** Photographs of eggs produced by different crosses. **(B)** Plot of the number of eggs hatched (gray) and unhatched (white) resulting from different crosses. **(C)** Morphologies of sperm in bursa copulatrix from representative control mating and from a representative WT female mated with Δ*BmSer1* mutant male. The data shown are means ± SEM (*n* = 30 pairs per group). Asterisks indicate significant differences with a two-tailed *t*-test: ^***^P < 0.001, n.s. not significant.

Progeny of WT virgin females crossed with WT males (control) and progeny of Δ*BmSer1* virgin females crossed with WT males grew and hatched normally. In contrast, WT or Δ*BmSer1* virgin females mated with Δ*BmSer1* males produced normal numbers of eggs, but these eggs did not hatch within 10 days (Figures 4A,B). We counted the number of eggs produced in broods by WT virgins sib-mated with Δ*BmSer1* and by Δ*BmSer1* virgins mated with WT males. These numbers were not significantly different compared with eggs produced by controls (Figure 4B). Δ*BmSer1* virgins mated with WT males produced a mean of 330 eggs, WT virgins mated with Δ*BmSer1* mutant males produced a mean of 318 eggs, Δ*BmSer1* virgin females mated with Δ*BmSer1* males produced a mean of 282 eggs, and WT females mated with WT males as a control produced a mean of 362 eggs (*n* = 30 pairs per group). Almost all control eggs hatched (∼93%, 336/362) as did eggs of Δ*BmSer1* virgins mated with WT males (∼82%, 269/330), but very few of the eggs of WT females mated with Δ*BmSer1* males hatched (∼6%, 19/318), and no eggs produced by Δ*BmSer1* virgins mated with Δ*BmSer1* males hatched (0%, 0/282) (Figure 4B). In addition, we observed sperm in the bursa copulatrix and found that the eupyrene sperm bundles in the control group had dissociated into single sperm, whereas the eupyrene sperm bundles in the WT females mated with Δ*BmSer1* mutant males had not dissociated into single sperm (Figure 4C). These results suggest that infertility of Δ*BmSer1* males is caused by the eupyrene sperm bundle dysfunction.

### Mutations Do Not Affect Adult Competitiveness and Are Heritable

**FIGURE 5:**
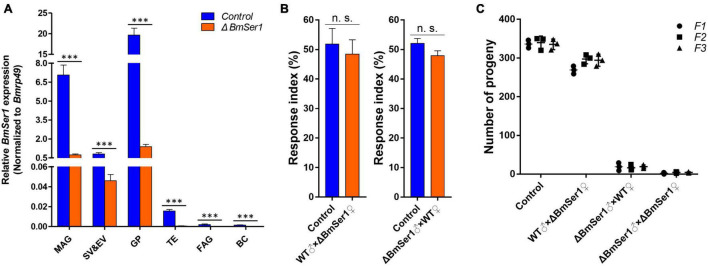
Competitiveness and heritability of *BmSer1* mutants. **(A)** Relative mRNA expression of *BmSer1* in six reproductive tissues of virgin adults. Abbreviations: male accessory gland (MAG), seminal vesicle and ejaculatory vesicle (SV&EV), glandula prostatica (GP), testis (TE), female accessory gland (FAG), and bursa copulatrix (BC). The mRNA expression was normalized to *Bmrp49*. **(B)** Response index of Δ*BmSer1* vs. WT females and males. **(C)** Number of progeny with indicated mutations in F1 (circle), F2 (square), and F3 (triangle) generations. The data shown are means ± S.E.M. Asterisks indicate significant differences with a two-tailed *t*-test: ^***^P < 0.001, n.s. no significant.

We next quantified expression of *BmSer1* mRNA in six major reproductive tissues of virgin WT and Δ*BmSer1* males. *BmSer1* was significantly down-regulated in all tissues evaluated in Δ*BmSer1* males compared with WT males (Figure 5A). To evaluate adult competitiveness, the response index was determined. This index is the percentage of successful matings relative to the total trials in a group. There was no significant difference in competitiveness between Δ*BmSer1* and WT females (control, 51.85%; female mutant, 48.48%; *n* = 30 per group) nor was there any significant difference in male competitiveness (control, 52.09%; male mutant, 47.91%; *n* = 30 per group) (Figure 5B).

As germline transformants were constructed by transgenic CRISPR/Cas9 technology, the mutations in *BmSer1* should be transmitted to progeny. Four lines were produced by hybridization of the *Nos-Cas9* line with the *U6-sgRNA* lines, and random hybridization of the four lines also produced only these four types of individuals. Hatch rates of each generation were consistent with expected inheritance of the mutation, and the male mutants remained sterile (Figure 5C). These results demonstrated that male sterility induced by disruption of *BmSer1* with CRISPR/Cas9 was transmitted into the next generation.

## Discussion

In most tissues, the expression of *BmSer1* was higher during the W stage than in the L5D3 stage, especially in gonads. Subsequently, we observed *BmSer1* expression in the gonads of the adult stage and found that it was highly expressed in the male gonads, especially in the glandula prostatica. This may be relevant to the male reproductive success. For example, serine proteases are hydrolytic enzymes involved in spermatogenesis in testis, sperm capacitation in uterus, migration in female reproductive tract, recognition and binding with the zona pellucida on the egg surface, the acrosome reaction, and fusion of sperm and egg (Zhu et al., 2021). Here, we demonstrated the importance of the male-specific expression of serine protease encoded by *BmSer1* in male reproductive success by CRISPR/Cas9 in the silkworm.

Mutations in the *BmSer1 gene* caused abnormal eupyrene sperm bundle dissociation and male infertility, although mutant females were fertile. Sperm dichotomy is a unique characteristic of lepidoptera species (Phillips, 1971; Karr and Walters, 2015). The male transports eupyrene sperm bundles to the bursa copulatrix of female through ejaculation. About 30 min after entering the bursa copulatrix, the eupyrene sperm bundles dissociate into single curvilinear sperm that are capable of fusion with an egg (Chen et al., 2020). Spermatogenesis and morphology are normal in Δ*BmSer1*, but the loss of function of the *BmSer1* gene led to the failure of eupyrene sperm bundle dissociation.

SIT has proven to be a valuable approach for environmentally friendly pest control. The ideal SIT results from mutation of a gene that only affects reproduction without adversely impacting growth and development (Cong et al., 2013; Hsu et al., 2014). Further, male sterility is superior to female sterility, as this allows females to carry a factor that causes males to produce infertile offspring. When the mutant females are released into the wild, they mate with wild-type males. The female offspring of these matings can mate with wild-type males and produce offspring, whereas males can mate with wild-type females but no offspring are produced. In this way, mutant females spread, reducing the population (Ant et al., 2012; Labbe et al., 2012; Hammond et al., 2016; Marubbi et al., 2017). Mutation of the *BmSer1* gene did not affect the growth and development of either sex, and male competitiveness and female attractiveness were no different from those of the wild-type adults. We also found that male sterility due to the mutation in *BmSer1* was stably passed through generations by females carrying the mutant gene, and male mutants were sterile in each generation. Therefore, the *Ser1* gene is a potential target for SIT.

In summary, mutations of the *Ser1* genes in *B. mori* resulted in male sterility, likely due to a defect in the dissociation process of eupyrene sperm bundles. Phylogenetic sequence analysis showed that the proteins encoded by the *Ser1* genes are moderately conserved in Lepidoptera. Lepidoptera, the second largest insect order, includes more than 70% of agroforestry pests (Roscoe et al., 2016). Thus, our study demonstrates that the *Ser1* gene is a potential molecular target for genetic-based pest management in a variety of Lepidoptera. The ideal SIT target gene is the one that can be mutated without altering viability or competitiveness of individuals but that causes male sterility (Chen et al., 2016; Collins, 2018). The *Ser1* mutation causes male sterility without affecting other growth indicators, therefore, it is a suitable target gene for biological pest control.

## Data Availability Statement

The original contributions presented in the study are included in the article/supplementary material, further inquiries can be directed to the corresponding author/s.

## Author Contributions

XX, YQW, and YH designed the research. XX and YHW performed the experiment and wrote the manuscript. JC, XD, and LY analyzed the data. JX and YZ revised and improved the manuscript. YQW and YH gave the final approval of the manuscript. All authors have approved the final version of the manuscript.

## Conflict of Interest

The authors declare that the research was conducted in the absence of any commercial or financial relationships that could be construed as a potential conflict of interest.

## Publisher’s Note

All claims expressed in this article are solely those of the authors and do not necessarily represent those of their affiliated organizations, or those of the publisher, the editors and the reviewers. Any product that may be evaluated in this article, or claim that may be made by its manufacturer, is not guaranteed or endorsed by the publisher.

## Footnotes

1
https://swissmodel.expasy.org/

2
http://crispr.dbcls.jp/

## References

[B1] Ant T. Koukidou M. Rempoulakis P. Gong H. F. Economopoulos A. Vontas J. (2012). Control of the olive fruit fly using genetics-enhanced sterile insect technique. *BMC Biol*. 10:51. 10.1186/1741-7007-10-51 22713628 PMC3398856

[B2] Barzkar N. Khan Z. Tamadoni Jahromi S. Pourmozaffar S. Gozari M. Nahavandi R. (2021). A critical review on marine serine protease and its inhibitors: a new wave of drugs? *Int. J. Biol. Macromol*. 170 674–687. 10.1016/j.ijbiomac.2020.12.134 33387547

[B3] Betzel C. Gourinath S. Kumar P. Kaur P. Perbandt M. Eschenburg S. (2001). Structure of a serine protease proteinase K from Tritirachium album limber at 0.98 A resolution. *Biochemistry* 40 3080–3088. 10.1021/bi002538n 11258922

[B4] Bhuiyan M. S. Fukunaga K. (2008). Activation of HtrA2, a mitochondrial serine protease mediates apoptosis: current knowledge on HtrA2 mediated myocardial ischemia/reperfusion injury. *Cardiovasc. Ther*. 26 224–232. 10.1111/j.1755-5922.2008.00052.x 18786092

[B5] Bienert S. Waterhouse A. De Beer T. A. Tauriello G. Studer G. Bordoli L. (2017). The SWISS-MODEL repository-new features and functionality. *Nucleic Acids Res*. 45 313–319. 10.1093/nar/gkw1132 27899672 PMC5210589

[B6] Cesari A. Monclus Mde L. Tejon G. P. Clementi M. Fornes M. W. (2010). Regulated serine proteinase lytic system on mammalian sperm surface: there must be a role. *Theriogenology* 74 699–711. 10.1016/j.theriogenology.2010.03.029 20537374

[B7] Chen L. Wang G. Zhu Y. N. Xiang H. Wang W. (2016). Advances and perspectives in the application of CRISPR/Cas9 in insects. *Dongwuxue Yanjiu* 37 220–228. 10.13918/j.issn.2095-8137.2016.4.220 27469253 PMC4978943

[B8] Chen S. Liu Y. Yang X. Liu Z. Luo X. Xu J. (2020). Dysfunction of dimorphic sperm impairs male fertility in the silkworm. *Cell Discov*. 6:60. 10.1038/s41421-020-00194-6 32963806 PMC7477584

[B9] Collins J. P. (2018). Gene drives in our future: challenges of and opportunities for using a self-sustaining technology in pest and vector management. *BMC Proc*. 12:9. 10.1186/s12919-018-0110-4 30079101 PMC6069294

[B10] Cong L. Ran F. A. Cox D. Lin S. Barretto R. Habib N. (2013). Multiplex genome engineering using CRISPR/Cas systems. *Science* 339 819–823. 10.1126/science.1231143 23287718 PMC3795411

[B11] Giorgino T. (2019). Analysis libraries for molecular trajectories: a cross-language synopsis. *Methods Mol. Biol*. 2022 503–527. 10.1007/978-1-4939-9608-7_20 31396916

[B12] Gyamera-Acheampong C. Mbikay M. (2009). Proprotein convertase subtilisin/kexin type 4 in mammalian fertility: a review. *Hum. Reprod. Update* 15 237–247. 10.1093/humupd/dmn060 19109312

[B13] Hammond A. Galizi R. Kyrou K. Simoni A. Siniscalchi C. Katsanos D. (2016). A CRISPR-Cas9 gene drive system targeting female reproduction in the malaria mosquito vector *Anopheles gambiae*. *Nat. Biotechnol*. 34 78–83. 10.1038/nbt.3439 26641531 PMC4913862

[B14] Holcomb R. J. Oura S. Nozawa K. Kent K. Yu Z. Robertson M. J. (2020). The testis-specific serine proteases PRSS44, PRSS46, and PRSS54 are dispensable for male mouse fertility. *Biol. Reprod*. 102 84–91. 10.1093/biolre/ioz158 31403672 PMC7013879

[B15] Hsu P. D. Lander E. S. Zhang F. (2014). Development and applications of CRISPR-Cas9 for genome engineering. *Cell* 157 1262–1278. 10.1016/j.cell.2014.05.010 24906146 PMC4343198

[B16] Karr T. L. Walters J. R. (2015). Panning for sperm gold: isolation and purification of apyrene and eupyrene sperm from lepidopterans. *Insect Biochem. Mol. Biol*. 63 152–158. 10.1016/j.ibmb.2015.06.007 26141489

[B17] Kasafirek E. Fric P. Slaby J. Malis F. (1976). p-Nitroanilides of 3-carboxypropionyl-peptides. Their cleavage by elastase, trypsin, and chymotrypsin. *Eur. J. Biochem* 69 1–13. 10.1111/j.1432-1033.1976.tb10852.x 991849

[B18] Kobayashi K. Endo T. Matsumura T. Lu Y. Yu Z. Matzuk M. M. (2020). Prss55 but not Prss51 is required for male fertility in mice. *Biol. Reprod*. 103 223–234. 10.1093/biolre/ioaa041 32301961 PMC7401375

[B19] Kraut J. (1977). Serine proteases: structure and mechanism of catalysis. *Annu. Rev. Biochem*. 46 331–358. 10.1146/annurev.bi.46.070177.001555 332063

[B20] Kumar S. Stecher G. Li M. Knyaz C. Tamura K. (2018). MEGA X: molecular evolutionary genetics analysis across computing platforms. *Mol. Biol. Evol*. 35 1547–1549. 10.1093/molbev/msy096 29722887 PMC5967553

[B21] Labbe G. M. Scaife S. Morgan S. A. Curtis Z. H. Alphey L. (2012). Female-specific flightless (fsRIDL) phenotype for control of *Aedes albopictus*. *PLoS Negl. Trop. Dis*. 6:e1724. 10.1371/journal.pntd.0001724 22802980 PMC3393675

[B22] LaFlamme B. A. Wolfner M. F. (2013). Identification and function of proteolysis regulators in seminal fluid. *Mol. Reprod. Dev*. 80 80–101. 10.1002/mrd.22130 23109270 PMC3923310

[B23] LaFlamme B. A. Ram K. R. Wolfner M. F. (2012). The *Drosophila melanogaster* seminal fluid protease “seminase” regulates proteolytic and post-mating reproductive processes. *PLoS Genet*. 8:e1002435. 10.1371/journal.pgen.1002435 22253601 PMC3257295

[B24] Le Magueresse-Battistoni B. (2007). Serine proteases and serine protease inhibitors in testicular physiology: the plasminogen activation system. *Reproduction* 134 721–729. 10.1530/REP-07-0114 18042629

[B25] Lee K. S. Kim B. Y. Choo Y. M. Jin B. R. (2018). Dual role of the serine protease homolog BmSPH-1 in the development and immunity of the silkworm *Bombyx mori*. *Dev. Comp. Immunol*. 85 170–176. 10.1016/j.dci.2018.04.011 29684723

[B26] Lin H. Xia X. Yu L. Vasseur L. Gurr G. M. Yao F. (2015). Genome-wide identification and expression profiling of serine proteases and homologs in the diamondback moth, *Plutella xylostella* (L.). *BMC Genomics* 16:1054. 10.1186/s12864-015-2243-4 26653876 PMC4676143

[B27] Liu J. Shen C. Fan W. Chen Y. Zhang A. Feng Y. (2016). Low levels of PRSS37 protein in sperm are associated with many cases of unexplained male infertility. *Acta Biochim. Biophys. Sin. (Shanghai)* 48 1058–1065. 10.1093/abbs/gmw096 27649891

[B28] Liu N. Phillips T. Zhang M. Wang Y. Opferman J. T. Shah R. (2004). Serine protease inhibitor 2A is a protective factor for memory T cell development. *Nat. Immunol*. 5 919–926. 10.1038/ni1107 15311278

[B29] Liu Y. X. Liu X. M. Nin L. F. Shi L. Chen S. R. (2013). Serine protease and ovarian paracrine factors in regulation of ovulation. *Front. Biosci. (Landmark Ed)* 18 650–664. 10.2741/4128 23276950

[B30] Marubbi T. Cassidy C. Miller E. Koukidou M. Martin-Rendon E. Warner S. (2017). Exposure to genetically engineered olive fly (*Bactrocera oleae*) has no negative impact on three non-target organisms. *Sci. Rep*. 7:11478. 10.1038/s41598-017-11908-4 28904391 PMC5597591

[B31] Naito Y. Hino K. Bono H. Ui-Tei K. (2015). CRISPRdirect: software for designing CRISPR/Cas guide RNA with reduced off-target sites. *Bioinformatics* 31 1120–1123. 10.1093/bioinformatics/btu743 25414360 PMC4382898

[B32] Nakao H. Matsumoto T. Oba Y. Niimi T. Yaginuma T. (2008). Germ cell specification and early embryonic patterning in *Bombyx mori* as revealed by nanos orthologues. *Evol. Dev*. 10 546–554. 10.1111/j.1525-142X.2008.00270.x 18803773

[B33] Nayyab S. Gervasi M. G. Tourzani D. A. Caraballo D. A. Jha K. N. Teves M. E. (2021). TSSK3, a novel target for male contraception, is required for spermiogenesis. *Mol. Reprod. Dev*. 88 718–730. 10.1002/mrd.23539 34623009 PMC8961454

[B34] Page M. J. Di Cera E. (2008). Serine peptidases: classification, structure and function. *Cell. Mol. Life Sci*. 65 1220–1236. 10.1007/s00018-008-7565-9 18259688 PMC11131664

[B35] Phillips D. M. (1971). Morphogenesis of the lacinate appendages of lepidopteran spermatozoa. *J. Ultrastruct. Res*. 34 567–585. 10.1016/s0022-5320(71)80064-3 5555018

[B36] Roscoe L. E. Silk P. Eveleigh E. S. (2016). Evidence of male hair pencil pheromone in *Choristoneura fumiferana* (Lepidoptera: Tortricidae). *J. Insect Sci*. 16:27. 10.1093/jisesa/iew010 26945090 PMC4782507

[B37] Saitou N. Nei M. (1987). The neighbor-joining method: a new method for reconstructing phylogenetic trees. *Mol. Biol. Evol*. 4 406–425. 10.1093/oxfordjournals.molbev.a040454 3447015

[B38] Salicioni A. M. Gervasi M. G. Sosnik J. Tourzani D. A. Nayyab S. Caraballo D. A. (2020). Testis-specific serine kinase protein family in male fertility and as targets for non-hormonal male contraception. *Biol. Reprod*. 103 264–274. 10.1093/biolre/ioaa064 32337545 PMC7401350

[B39] Shang X. Shen C. Liu J. Tang L. Zhang H. Wang Y. (2018). Serine protease PRSS55 is crucial for male mouse fertility via affecting sperm migration and sperm-egg binding. *Cell. Mol. Life Sci*. 75 4371–4384. 10.1007/s00018-018-2878-9 30032357 PMC6208766

[B40] Tan A. Tanaka H. Tamura T. Shiotsuki T. (2005). Precocious metamorphosis in transgenic silkworms overexpressing juvenile hormone esterase. *Proc. Natl. Acad. Sci. U.S.A*. 102 11751–11756. 10.1073/pnas.0500954102 16087883 PMC1187958

[B41] Tardif S. Guyonnet B. Cormier N. Cornwall G. A. (2012). Alteration in the processing of the ACRBP/sp32 protein and sperm head/acrosome malformations in proprotein convertase 4 (PCSK4) null mice. *Mol. Hum. Reprod*. 18 298–307. 10.1093/molehr/gas009 22357636 PMC3358042

[B42] Wallace A. C. Laskowski R. A. Thornton J. M. (1996). Derivation of 3D coordinate templates for searching structural databases: application to Ser-His-Asp catalytic triads in the serine proteinases and lipases. *Protein Sci*. 5 1001–1013. 10.1002/pro.5560050603 8762132 PMC2143436

[B43] Wang J. Ohmuraya M. Hirota M. Baba H. Zhao G. Takeya M. (2008). Expression pattern of serine protease inhibitor kazal type 3 (Spink3) during mouse embryonic development. *Histochem. Cell Biol*. 130 387–397. 10.1007/s00418-008-0425-8 18386042

[B44] Xiong W. Shen C. Li C. Zhang X. Ge H. Tang L. (2021). Dissecting the PRSS37 interactome and potential mechanisms leading to ADAM3 loss in PRSS37-null sperm. *J. Cell Sci*. 134:jcs258426. 10.1242/jcs.258426 34028541

[B45] Xu J. Chen R. M. Chen S. Q. Chen K. Tang L. M. Yang D. H. (2019). Identification of a germline-expression promoter for genome editing in *Bombyx mori*. *Insect Sci.* 26 991–999. 10.1111/1744-7917.12657 30549429

[B46] Xu X. Bi H. Wang Y. Li X. Xu J. Liu Z. (2020b). Disruption of the *ovarian serine protease* (*Osp*) gene causes female sterility in *Bombyx mori* and *Spodoptera litura*. *Pest Manag. Sci*. 76 1245–1255. 10.1002/ps.5634 31595658

[B47] Xu X. Wang Y. Bi H. Xu J. Liu Z. Niu C. (2020c). Mutation of the seminal protease gene, *serine protease 2*, results in male sterility in diverse lepidopterans. *Insect Biochem. Mol. Biol*. 116:103243. 10.1016/j.ibmb.2019.103243 31541694

[B48] Xu J. Liu W. Yang D. Chen S. Chen K. Liu Z. (2020a). Regulation of olfactory-based sex behaviors in the silkworm by genes in the sex-determination cascade. *PLoS Genet*. 16:e1008622. 10.1371/journal.pgen.1008622 32520935 PMC7307793

[B49] Xu X. Wang Y. H. Liu Z. L. Wang Y. Q. He L. Li K. (2021). Disruption of egg-specific protein causes female sterility in *Bombyx mori*. *Insect Sci*. 10.1111/1744-7917.12904 [Epub ahead of print]. 33629486

[B50] Zhou G. W. Guo J. Huang W. Fletterick R. J. Scanlan T. S. (1994). Crystal structure of a catalytic antibody with a serine protease active site. *Science* 265 1059–1064. 10.1126/science.8066444 8066444

[B51] Zhu F. Li W. Zhou X. Chen X. Zheng M. Cui Y. (2021). PRSS55 plays an important role in the structural differentiation and energy metabolism of sperm and is required for male fertility in mice. *J. Cell. Mol. Med*. 25 2040–2051. 10.1111/jcmm.16116 33417308 PMC7882947

